# Polyphenols with Anti-Amyloid β Aggregation Show Potential Risk of Toxicity Via Pro-Oxidant Properties

**DOI:** 10.3390/ijms21103561

**Published:** 2020-05-18

**Authors:** Hatasu Kobayashi, Mariko Murata, Shosuke Kawanishi, Shinji Oikawa

**Affiliations:** 1 Department of Environmental and Molecular Medicine, Mie University Graduate School of Medicine, Tsu, Mie 514-8507, Japan; hatasuk@doc.medic.mie-u.ac.jp (H.K.); mmurata@doc.medic.mie-u.ac.jp (M.M.); 2 Faculty of Pharmaceutical Sciences, Suzuka University of Medical Science, Suzuka, Mie 513-8670, Japan; kawanisi@suzuka-u.ac.jp

**Keywords:** Amyloid β, Polyphenol, Pro-oxidant, Alzheimer’s Disease

## Abstract

Alzheimer’s disease (AD) is the most common form of dementia among older people. Amyloid β (Aβ) aggregation has been the focus for a therapeutic target for the treatment of AD. Naturally occurring polyphenols have an inhibitory effect on Aβ aggregation and have attracted a lot of attention for the development of treatment strategies which could mitigate the symptoms of AD. However, considerable evidence has shown that the pro-oxidant mechanisms of polyphenols could have a deleterious effect. Our group has established an assay system to evaluate the pro-oxidant characteristics of chemical compounds, based on their reactivity with DNA. In this review, we have summarized the anti-Aβ aggregation and pro-oxidant properties of polyphenols. These findings could contribute to understanding the mechanism underlying the potential risk of polyphenols. We would like to emphasize the importance of assessing the pro-oxidant properties of polyphenols from a safety point of view.

## 1. Amyloid β Aggregation in Alzheimer’s Disease

**Figure 1:**
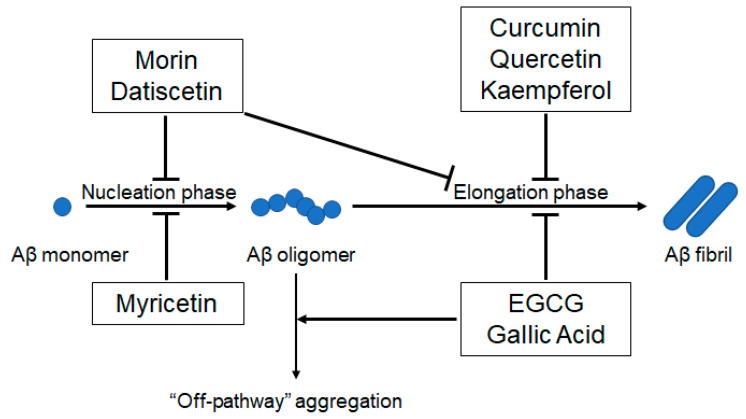
A schematic model showing the inhibitory effects of polyphenols on Aβ aggregation, based on the “amyloid cascade hypothesis.” Myricetin inhibits nucleation [8]. Morin and datiscetin inhibit nucleation and elongation [9]. Curcumin [10], quercetin [11], and kaempferol [9] inhibit elongation. EGCG [12] and gallic acid [13] inhibit elongation and redirect Aβ oligomers to “off-pathway” aggregation. Aβ: amyloid β, EGCG: epigallocatechin gallate.

Alzheimer’s disease (AD) is the leading cause of dementia, and disease prevalence has been increasing dramatically with a worldwide increase in the aging population [1]. Numerous studies have suggested that accumulation of the Amyloid β (Aβ) peptide in the brain is the initial pathological event for AD [2]. The Aβ peptide is a soluble, extracellular fragment generated from the sequential cleavage of the amyloid precursor protein (APP) by β- and γ-secretases [3]. Aβ accumulation promotes conformational changes in the peptide, resulting in the formation of oligomers and fibrils; ultimately, resulting in plaque deposition—one of the hallmarks of AD pathology [2,4]. The nucleation-dependent polymerization mechanism, which separates the amyloid fibrillization process into a nucleation phase and an elongation phase [5], is currently proposed as an aggregation mechanism for the Aβ peptide (Figure 1). During the nucleation phase, soluble Aβ monomers undergo conformational changes and self-associate to form oligomeric nuclei that are rich in β-sheets. During the elongation phase, these oligomeric nuclei act as a template and associate with monomers to initiate polymerization [6]. There are currently four approved medications for AD (three cholinesterase inhibitors and one uncompetitive NMDA receptor modulator), but they have a small effect size and show no effect on long-term disease progression [7]. Therefore, new drugs directed against various identified targets of AD, such as Aβ, tau, ApoE, and neuroinflammation are urgently needed [7]. Among these therapeutic targets, researchers have largely focused on Aβ aggregation for the prevention and treatment of AD, based on the “amyloid cascade hypothesis”.

## 2. Beneficial Anti-Aβ Aggregation and Adverse Pro-Oxidant Effects of Polyphenols

**Figure 2:**
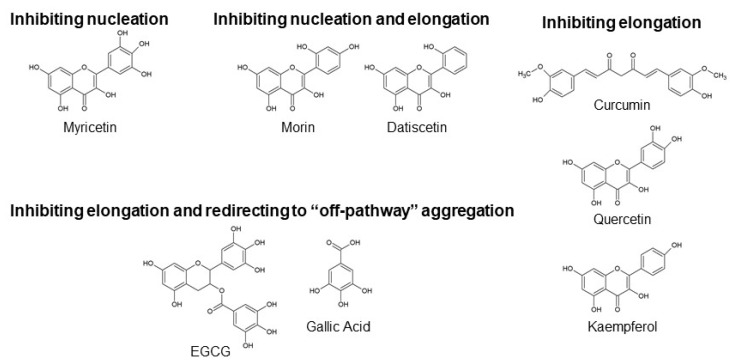
Chemical structures of polyphenols shown in Table 1. EGCG: epigallocatechin gallate

Researchers have investigated the inhibitory effects of various chemical and biological molecules on Aβ aggregation to develop a strategy for mitigating AD. These compounds include small organic molecules, peptide derivatives, chemical and molecular chaperones, and antibodies, to name a few [4]. Polyphenols are naturally occurring secondary metabolites found in large quantities in fruits, vegetables, seeds, and plant-derived oils; thus exhibit easy availability [14,15]. In vitro studies have shown that several polyphenols reduce Aβ aggregation by inhibiting the nucleation phase or elongation phase, or both, and redirecting the Aβ oligomers to the less-toxic “off-pathway” aggregation (Figure 1). Details of the anti-Aβ aggregation activity of each polyphenol are described below (Section 2.1–2.4). The anti-Aβ aggregation activities of some compounds have been confirmed in animal studies, and clinical studies have either been performed or are being performed to test these selected polyphenols [16,17]. However, considerable evidence has raised the concern that polyphenols could exert deleterious effects through their pro-oxidant mechanisms [18,19,20]. Many polyphenols involved in anti-Aβ aggregation have been reported to display pro-oxidant activities, which are potentially linked with toxic effects (Table 1, Figure 2). 

A common feature of polyphenols, especially those harboring hydroxyl groups in the phenol ring, is that they can readily participate in redox reactions [21], which is associated with both their antioxidant and pro-oxidant properties. Our group has established an assay to evaluate the pro-oxidant characteristics of chemical compounds on the basis of their ability to induce oxidative DNA damage, and investigated the mechanisms of the reactive oxygen species (ROS) generation [22]. Based on our results and that of others in the literature, in this review we have focused on polyphenols whose mechanisms of inhibiting Aβ aggregation have been well-studied, and have summarized their pro-oxidant properties. In addition, recent studies have suggested that biological activities of polyphenols were attributed to not only the polyphenols themselves but also their metabolites generated in vivo [23,24]. Therefore, we also have described some cases showing that metabolites are involved in pro-oxidant properties.

### 2.1. Polyphenols Involved in Inhibiting Nucleation

#### Myricetin

Myricetin is one of the most common naturally occurring compounds found in a large variety of plants and has been reported to show good biological activity as an antioxidant, anti-inflammatory, and anti-tumorigenic agent [64,65]. Studies using fluorescence spectroscopy with thioflavin T and electron microscopy have shown that myricetin inhibits the formation of Aβ fibrils [66]. Ono et al. demonstrated that myricetin blocked Aβ oligomer formation and bound to monomeric Aβ by an assay using a photoinduced cross-linking agent and nuclear magnetic resonance (NMR) [8]. These findings suggested that myricetin could prevent nucleation via direct binding to the Aβ monomer. Myricetin was also shown to reduce the number of high molecular weight oligomers and prevent the development of AD pathology in an AD mouse model [67].

Despite these encouraging results, myricetin has been reported to have mutagenic activity [32,33]. A recent study showed that myricetin tested positive in a bacterial mutagenicity assay and in vitro micronuclei formation assay [32]. Metal-mediated DNA damage induced by myricetin has been demonstrated in studies using plasmid DNA, isolated nuclei, and cultured cells [28,29,30,31]. The inhibitory effects of several ROS scavengers on DNA damage [28,31] and the generation of 8-oxo-7,8-dihydro-2′-deoxyguanosine (8-oxodG), an indicator of oxidative DNA damage [31], have indicated pro-oxidant mechanisms of myricetin-induced DNA damage.

### 2.2. Polyphenols Involved in Inhibiting Nucleation and Elongation

#### Morin and Datiscetin

**Figure 3:**
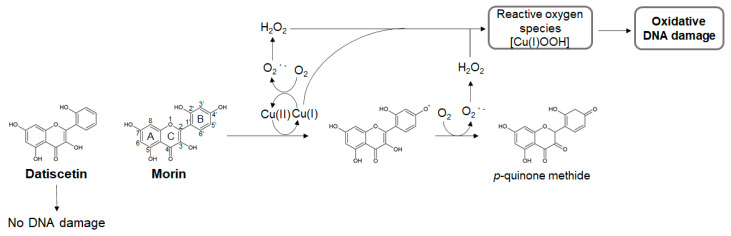
Possible mechanism of oxidative DNA damage induced by morin in the presence of Cu(II). The 4′-hydroxyl group of the B-ring of morin is responsible for the generation of Cu(I)-hydroperoxide (Cu(I)OOH) and the resultant oxidative DNA damage. Datiscetin, an analog of morin, without the 4′-hydroxyl group, does not damage DNA.

Morin a member of the flavonoid family, was originally isolated from the members of the *Moraceae* family and is found in a wide variety of fruits, vegetables, and herbs [68,69]. Morin, which has antioxidant and anti-inflammatory activities, has been reported to show pharmacological effects in several diseases [68,69,70]. The inhibitory effect of morin towards Aβ aggregation has been reported in several in vitro studies that tested naturally occurring compounds [66,71]. Furthermore, sustained treatment with morin could reduce the production of insoluble Aβ and the formation of amyloid plaques [72] and rescue cognitive impairment [72,73] in AD and dementia animal models. NMR analysis has shown that morin could prevent both the nucleation and the elongation phases during Aβ42 aggregation by interacting with His13, His14, and Gln15, which are close to the intermolecular β-sheet region of Aβ42 [9]. This anti-Aβ aggregation activity has been attributed to the C-1 oxygen of the C-ring and the 2′-hydroxyl group of the B-ring (Figure 3), which stabilize the flatness between the A-, B-, and C-rings of morin and enable it to interact with the intermolecular β-sheet region [74]. Datiscetin, which has the same structure as morin except for the 4′-hydroxyl group of the B-ring, also prevents Aβ aggregation by the same mechanism [9,74].

Previously, morin has been shown to promote ROS generation. Morin could induce metal-mediated lipid peroxidation of the nuclear membrane and DNA strand break in isolated nuclei [35]. The morin-Cu(II) complex could cleave plasmid DNA via an oxidative pathway [30,37]. Cell model studies have suggested that morin can cause DNA strand breaks though ROS production [34]. Morin was shown to have a mutagenic activity with the *Salmonella*/microsomal activation system [38]. Recently, we have shown that in the presence of Cu(II), morin induces not only DNA strand breaks but also base modification, including 8-oxodG formation, in isolated DNA [36]. By testing the effects of various ROS scavengers and Cu(I) chelators on DNA damage, we proposed that morin undergoes autoxidation via the Cu(I)/Cu(II) redox cycle, resulting in H_2_O_2_ generation to produce Cu(I)-hydroperoxide, which causes oxidative DNA damage (Figure 3) [36]. However, datiscetin, which lacks the 4′-hydroxyl group of the B-ring, did not induce DNA damage under our experimental condition (unpublished data). These results indicated that the 4′-hydroxyl group of the B-ring plays an important role in the pro-oxidant activity of morin.

### 2.3. Polyphenols Involved in Inhibiting Elongation

#### 2.3.1. Curcumin

Curcumin is the main naturally occurring polyphenol found in turmeric, which is isolated from the rhizome of *Curcuma longa* and is extensively used as a spice in curries and mustards [75]. Turmeric has also been traditionally used as a medicinal herb for the treatment of various diseases in Ayurvedic and traditional Chinese medicine [76]. Research on curcumin has shown that it possesses several protective and therapeutic properties, including anti-inflammatory, antioxidant, anti-microbial, and anti-cancer activity [77,78]. Recently, in the context of therapies for AD and other neurodegenerative diseases, Schubert et al. proposed a novel drug screening platform which finds candidates with multiple neuroprotective activities, and identified curcumin as a lead compound from the screening of natural product libraries [79]. As one of the neuroprotective properties, several in vitro [80,81] and in vivo [81,82] studies have demonstrated that curcumin can inhibit Aβ aggregation. Curcumin has been shown to prevent the formation and extension of Aβ fibrils and destabilize preformed Aβ fibrils in vitro [80]. Curcumin inhibits Aβ aggregation by directly binding Aβ to block its self-assembly in an in vitro aggregation assay [81]. NMR analysis has indicated that curcumin interacts with residue number 12 and 17–21, included in the β-sheet structure of the Aβ42 fibrils [10], suggesting that it has an inhibitory effect on fibril elongation. On the other hand, some researchers caution that these wide-range bioactivities of curcumin are characteristics of a pan-assay interference compound (PAINS) [83,84]. PAINS are compounds displaying activities which do not depend on a specific and drug-like interaction between molecule and protein, leading to artifact in multiple types of assays [83,84]. Thus, further research is needed to explore the therapeutic value of curcumin.

**Figure 4:**
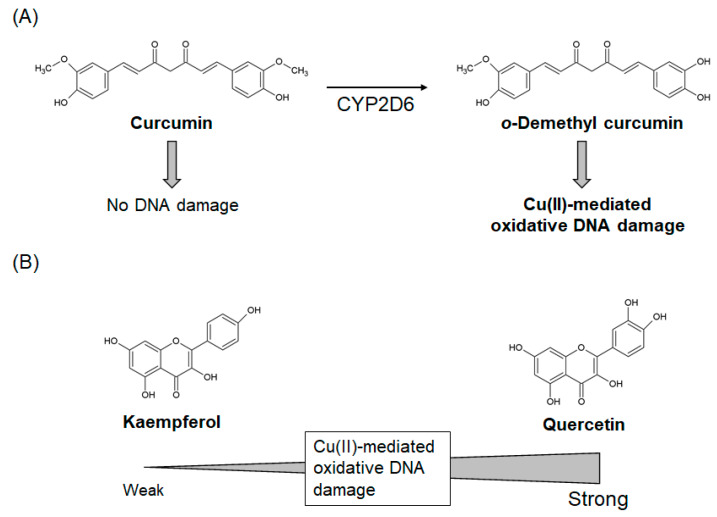
The role of catechol moieties in *o*-demethyl curcumin- and quercetin-mediated oxidative DNA damage in the presence of Cu(II). (**A**) *o*-Demethyl curcumin (with a catechol moiety) induced Cu(II)-mediated oxidative damage, while curcumin, its parent compound (without catechol moieties), did not. (**B**) Quercetin (with a catechol moiety) induced stronger oxidative damage than kaempferol, its analog (without catechol moieties) in the presence of Cu(II). CYP: cytochrome P450

Curcumin has also been reported to have pro-oxidant properties under some conditions [85]. Several studies have reported ROS generation and DNA damage in cultured cells exposed to curcumin [39,40,41]. A National Toxicology Program study has revealed that the dietary intake of turmeric oleoresin, which contains a high curcumin content (79–85%), induced hyperplasia of the colon mucosa in rats and increased hepatocellular adenoma in mice [43]. These findings raise the possibility that curcumin-induced oxidative DNA damage may promote tumorigenesis [78]. However, we have previously shown that curcumin does not cause damage to isolated DNA by itself, even in the presence of Cu(II) [42]. Curcumin/Cu(II)-mediated oxidative DNA damage has occurred only when curcumin was pre-treated with cytochrome P450 (CYP) enzymes, suggesting that metabolites of curcumin act as DNA-damaging agents [42]. Mass spectral analysis indicated CYP2D6-mediated *o*-demethyl curcumin formation, which was considered to generate Cu(I)-hydroperoxide during the autoxidation of *o*-demethyl curcumin, resulting in DNA damage [42]. CYP enzymes are known to be related to bioactivation of several chemical carcinogens [86], suggesting that curcumin-mediated hepatocellular adenoma in mice [43] might be explained by particular metabolisms of hepatocytes. *o*-demethyl curcumin has a catechol moiety (phenol with two hydroxy groups in the ortho-position), resulting from CYP-mediated demethylation of curcumin, that is likely to play a critical role in oxidative damage (Figure 4A). Thus, some antioxidants could be converted to pro-oxidants in particular metabolic conditions. These findings suggest that it is important to evaluate not only target polyphenols but also their metabolites.

#### 2.3.2. Quercetin and Kaempferol

Quercetin, a readily available naturally occurring polyphenol, is found abundantly in vegetables and fruits, such as onions and apples [87]. Quercetin is well known to have antioxidant and anti-inflammatory properties, and is expected to play protective roles in a wide range of diseases [88,89,90]. In vitro aggregation studies show that quercetin inhibits Aβ fibril formation by strengthening the hydrophobic interactions between the Aβ β-sheet structure and the aromatic ring by hydrogen bonding [91]. Quercetin exerts an anti-amyloidogenic effect in vitro by preferentially binding to Aβ fibrils at the growth edge, rather than to Aβ monomers, resulting in inhibition of fibril elongation [11]. Quercetin also reduces Aβ-induced neurotoxicity in a cell system overexpressing mutant APP, which is associated with early-onset familial AD [91]. Treatment with quercetin reduced the number and size of Aβ plaques and improves cognitive function in an AD mouse model [92]. Kaempferol, an analog of quercetin, also showed anti-Aβ aggregation activity [66]

Although the beneficial effects of quercetin are widely accepted, there is a concern about its potential pro-oxidant and cytotoxic activities when used therapeutically [93]. Results from several in vitro and in vivo studies suggest that quercetin has a pro-oxidant effect in addition to its antioxidant effect [34,94]. Quercetin has been reported to be mutagenic [47,48,49], and induce renal tubule adenocarcinomas [48] and intestinal and bladder cancer [50] in rats. We have previously shown that quercetin induced oxidative DNA damage both in isolated and cellular DNA [45,46]. Quercetin caused 8-oxodG formation in HL-60 cells, but not in their H_2_O_2_-resistant clones, HP100 cells, indicating that H_2_O_2_ is the main mediator of DNA damage and cytotoxicity in this context [45]. The pro-oxidant activity of quercetin is likely due to the presence of the catechol moiety and the resultant susceptibility to autoxidation, leading to conversion into ortho-semiquinone and ortho-quinone [95,96]. This finding is supported by the observation that kaempferol, a quercetin analog without catechol moieties, induces markedly weaker oxidative DNA damage than quercetin (Figure 4B) [46]. Furthermore, quercetin exhibits both mutagenicity and carcinogenicity [48,49,50], whereas kaempferol exhibits only mutagenicity (Table 1) [47], which might reflect the different extent of oxidative DNA damage caused by quercetin and kaempferol.

### 2.4. Polyphenols Involved in Inhibiting Elongation and Redirecting Aβ Monomers to “Off-Pathway” Aggregation

#### 2.4.1. Epigallocatechin Gallate (EGCG) and Other Green Tea Catechins

Numerous epidemiological studies have demonstrated that consumption of green tea has many health benefits [97]. Among green tea catechins, EGCG is most abundant (65% of the total catechin content in green tea) and most biologically active [98]. EGCG is a powerful antioxidant, anti-inflammatory, and anti-infective agent, and is suggested to have protective effects in fighting many diseases [99,100,101]. EGCG inhibits Aβ fibrillogenesis by directly binding to natively unfolded polypeptides and promoting the formation of unstructured and nontoxic oligomers (so-called “off-pathway” aggregation) instead of toxic β-sheet–rich fibril [12,102]. EGCG oxidation products, such as quinones, may be involved in redirecting “off-pathway” aggregation by covalently binding to lysine of Aβ through a Schiff base formation [103]. In vitro studies have also demonstrated the ability of EGCG to convert mature Aβ fibrils into “off-pathway” aggregation by directly binding to the β-sheet-rich fibril and mediating conformational change [104]. In addition, Rezai-Zadeh et al. have reported that EGCG treatment decreases the Aβ plaque burden in the brain and improves working memory, using an AD mouse model [105,106].

**Figure 5:**
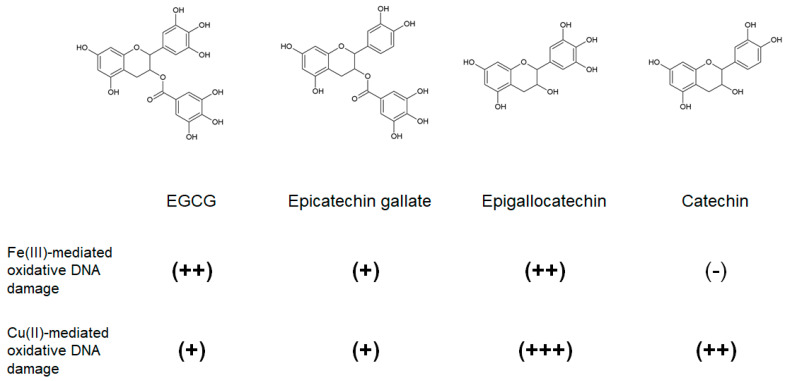
Fe(III)- and Cu(II)-mediated DNA damage caused by green tea polyphenols. Three tea polyphenols with pyrogallol moieties (EGCG, epicatechin gallate, and epigallocatechin) can induce Fe(III)- and Cu(II)-mediated oxidative DNA damage although, catechins, which harbor no pyrogallol moieties, only cause Cu(II)-mediated oxidative DNA damage. (+++), (++), (+), and (-) represent the extent of DNA damage. EGCG: epigallocatechin gallate

However, many reports have suggested links between intake of high dose of EGCG and damage in several organs, especially the liver, in humans [56,107,108]. In 2018, the European Food Safety Authority concluded that intake of doses equal or above 800 mg EGCG/day, taken as a food supplement, can induce a significant increase of serum transaminases, which is indicative of liver injury [107]. A National Toxicology Program study reported that oral administration of green tea extracts containing EGCG (48.4% by weight) induced lesions in the gastrointestinal tract and liver in rats and mice [55]. A few cell model studies have shown that EGCG induces cellular DNA damage [53,109]. These potential harmful effects of EGCG have been attributed to its pro-oxidant activity [110,111,112]. Our previous report has indicated that EGCG significantly increases the content of 8-oxodG of DNA in cultured cells, but not in its H_2_O_2_-resistant clone cell [54], which is consistent with studies demonstrating intracellular ROS generation in cultured cells treated with EGCG [113,114]. Furthermore, EGCG caused oxidative damage to isolated DNA in the presence of Fe(III) and Cu(II) [54]. This was likely due to the generation of different ROS: hydroxy radical from the reaction of Fe(II) with H_2_O_2_ and Cu(I)-hydroperoxide from the reaction of Cu(I) with H_2_O_2_ [54]. To investigate the association between the chemical structure of green tea polyphenols and metal-mediated ROS generation, we compared EGCG-induced oxidative DNA damage in the presence of Fe(III) and Cu(II) with epicatechin gallate [115], epigallocatechin [116] and catechin [116], which are the other main green tea polyphenols that exert anti-Aβ aggregation activities. The results showed that EGCG, epicatechin gallate and epigallocatechin induced oxidative DNA damage in the presence of Fe(III) and Cu(II), whereas catechin did so in the presence of Cu(II) alone [54], suggesting that the pyrogallol moiety (phenolic three hydroxyl group) may be critical for Fe(III)-mediated ROS generation in green tea catechins (Figure 5).

#### 2.4.2. Propyl Gallate and Gallic Acid

Propyl gallate and gallic acid have been reported to inhibit Aβ aggregation [117]. The anti-Aβ aggregation activities of gallic acid have been well-studied; the mechanism by which propyl gallate inhibits Aβ aggregation remains unknown. Gallic acid is an abundantly found polyphenol in the plant kingdom and is present in tea, wine, and fruits, such as grape and berries [118]. Gallic acid has been reported to have a beneficial effect on health and is pharmacologically effective in many diseases [119]. In relation to AD, several in vitro studies have demonstrated that gallic acid can reduce Aβ aggregation [13,117,120,121]. Molecular docking studies have shown that gallic acid interacts with Aβ aggregates and inhibits Aβ fibril formation by disrupting the Lys28-Ala42 salt bridge of Aβ [13]. Alternatively, gallic acid may convert toxic Aβ aggregates into “off-pathway” aggregation [122], similar to previously reported properties of EGCG [12,102]. Recently, Yu et al. have reported that gallic acid treatment alleviates cognitive decline in an AD mouse model at both early and late stages [13].

**Figure 6:**
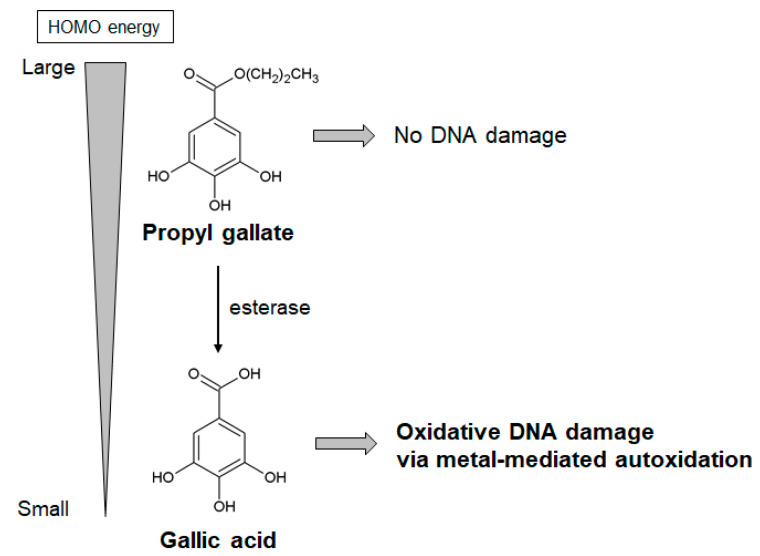
Estimation of HOMO energy to assess the pro-oxidant reactivity of gallic acid and propyl gallate. HOMO energies of gallic acid and propyl gallate were estimated from ab initio molecular orbital calculations at Hartree–Fock 6-31G* level. Calculations were performed using Spartan 02 for Windows (Wavefunction Inc., CA) [125]. HOMO energy: highest occupied molecular orbital energy

In contrast, potential harmful effects of propyl gallate and gallic acid, associated with their pro-oxidant properties, have also been reported [59,60,61,62,63]. Propyl gallate, but not gallic acid, is carcinogenic in mice and rats [123]. While propyl gallate led to 8-oxodG formation in cultured cells, it did not induce damage in isolated DNA [60]. Propyl gallate has been known to convert to gallic acid by an esterase (Figure 6) [124]. Therefore, to clarify its mechanism of carcinogenicity, we studied isolated DNA damage caused by gallic acid. Gallic acid and esterase-treated propyl gallate could induce Fe(III)- and Cu(II)-dependent oxidative DNA damage in isolated DNA through metal-mediated autoxidation [60]. These results suggest that gallic acid converted from propyl gallate plays an important role in propyl gallate-mediated carcinogenicity. To understand why gallic acid, but not propyl gallate, induces oxidative DNA damage, highest occupied molecular orbital (HOMO) energy estimation [125] was performed. The HOMO energy of the anionic form of gallic acid is smaller than that of propyl gallate, suggesting that gallic acid can readily undergo autoxidation compared to propyl gallate (Figure 6) [60]. Furthermore, gallic acid has been reported to display toxic effects other than carcinogenesis [59,62,63]. Administration of gallic acid induces liver injury [62,63] in mice and rats, and renal damage [63] in rats. ROS-associated cytotoxicity of gallic acid against noncancerous cell has been demonstrated using rat primary cultured hepatocytes [62] and vascular smooth muscle cells [59].

## 3. The Role of Phenolic Hydroxyl Groups in Anti-Aβ Aggregation and Pro-Oxidant Activities of Polyphenols

The phenolic hydroxyl groups of polyphenols are considered to be essential for its anti-Aβ aggregation activity. Quinones generated from phenolic hydroxyl groups can react with the lysine side chains of proteins [126]. Lys28 of Aβ has been reported to be critical for Aβ42 aggregation [127]. Therefore, quinones, especially catechol-type quinones, may contribute to the inhibition of Aβ aggregation. This is supported by the finding that the interactions between quinones from several polyphenols and lysine of Aβ play an important role in the inhibition of Aβ aggregation [103,128]. In contrast, our studies have shown ROS generation by several polyphenols through their autoxidation and quinone formation in the presence of metal ions such as Cu(II) [36,42,45,46,54,60,129]. In addition, some metabolites of target polyphenols also display pro-oxidant activities via quinone formation, even though target polyphenols themselves are not pro-oxidant [42,60].

Some studies have reported binding of the phenolic hydroxyl groups with histidine in anti-amyloid aggregation activity [9,130]. Histidine residues of Aβ impact Aβ aggregation by affecting the oligomeric equilibria [131] and interacting with metal ions [132]. Morin interacts with His13, His14, and Gln15 of Aβ42, corresponding to the intermolecular regions of β-sheets, and prevents Aβ assembly likely via its aromatic rings [9]. In the case of islet amyloid polypeptide, curcumin was shown to prevent inter-peptide interaction between Phe15 and His18, which is important for the aggregation of amyloids [130]. However, we have suggested that phenolic hydroxyl groups of morin and a metabolite of curcumin react with Cu(II), which leads to ROS generation and oxidative DNA damage [36,42].

Interestingly, copper is also thought to be associated with the enhancement of Aβ aggregation. The level of copper is elevated in the blood of AD patients [133] and Aβ plaques in an AD mouse model [134]. Cu(II) interacts with Aβ and enables the formation of β-sheets via its binding to His13 and His14, thereby forming a brace between Aβ strands [135]. Several polyphenols enable the chelating of various metal ions [136,137]. A recent report has shown that EGCG inhibits Cu(II)-associated amyloid aggregation of α-synuclein [138]. These findings suggest that polyphenols may inhibit Aβ aggregation via a Cu(II) chelating mechanism. However, as mentioned above, the interaction of polyphenols with Cu(II) leads to concomitant oxidative DNA damage [36,42,45,46,54,60,129].

These findings suggest that polyphenols can block Aβ aggregation and cause oxidative damage under certain circumstances, such as when they are in proximity to DNA.

## 4. Conclusions

Naturally occurring polyphenols are generally regarded as safe, based on their long history of use in the diet. However, when used at pharmacological concentrations, they have potential risks [18,19,20]. In this review, the pro-oxidant properties and the associated toxic effects of several naturally occurring polyphenols with anti-Aβ aggregation activity have been summarized. The pro-oxidant and anti-Aβ aggregation effects can be attributed to the structural features of polyphenols, suggesting a potential risk of oxidative damage. Therefore, we would like to emphasize the importance of assessing pro-oxidant properties of polyphenols from the point of view of safety.

## Figures and Tables

**Table 1:** Toxic effects associated with pro-oxidant properties of naturally occurring polyphenols harboring anti-Aβ aggregation activity.

Anti-Aβ Aggregation Effect	Polyphenol	Toxic Effects Associated with Pro-Oxidant Properties	Concentration or Dose Showing Toxic Effects of Polyphenols
**Inhibiting nucleation**	Myricetin	**Cytotoxicity**	
		Cytotoxicity linked with ROS generation	Cell: 20 μM [25], 50 μM [26,27]
		**Genotoxicity**	
		Oxidative DNA damage	Cell: 20 μM [28], 50 μM [29] DNA: 5 μM [30], 200 μM [31]
		Mutagenic activity	Bacteria: 0.628 μmol/plate [32] Cell: 42 μM [33]
**Inhibiting nucleation and elongation**	Morin	**Genotoxicity**	
		Oxidative DNA damage	Cell: 100 μM [34] DNA: 5 μM [30],10 μM [35], 20 μM [36], 100 μM [37]
		Mutagenic activity	Bacteria: 0.149 μmol/plate [38]
	Datiscetin	No report	
**Inhibiting elongation**	Curcumin	**Cytotoxicity**	
		Cytotoxicity linked with ROS generation	Cell: 5 μM [39], 50 μM [40]
		**Genotoxicity**	
		DNA damage in cultured cell	Cell: 50 μM [41]
		Curcumin metabolite-mediated oxidative damage in isolated DNA	DNA: 2 μM [42]
		**Tumorigenicity**	
		Colon mucosal hyperplasia and hepatocellular adenoma in rats and mice treated with turmeric oleoresin containing curcumin (79%-85%), respectively	Colon hyperplasia: 2000 mg/kg/day (male rats) [43] Hepatocellular adenoma: 520 mg/kg/day (male mice) [43], 1620 mg/kg/day (female mice) [43]
**Inhibiting elongation** (continued)	Quercetin	**Cytotoxicity**	
		Cytotoxicity linked with ROS generation	Cell: 50 μM [44]
		**Genotoxicity**	
		Oxidative DNA damage	Cell: 30 μM [45], 50 μM [29], 100 μM [34] DNA: 10 μM [46]
		Mutagenic activity	Bacteria: 0.121 μmol/plate [47] Cell: 2.2 μM [48], 32.5 μM [49]
		**Carcinogenesis**	
		Renal tubule adenocarcinomas and intestinal and bladder cancer in rats	Renal tubule adenocarcinomas: 1900 mg/kg/day (male rats) [48] Intestinal and bladder cancer: 27.8 mM/rat (male, cumulative dose) [50], 25.3 mM/rat (female, cumulative dose) [50]
	Kaempferol	**Genotoxicity**	
		Oxidative DNA damage	Cell: 50 μM [29]
		Mutagenic activity	Bacteria: 0.143 μmol/plate [47]
**Inhibiting elongation and redirecting to “off-pathway” aggregation**	EGCG	**Cytotoxicity**	
		Cytotoxicity linked with ROS generation	Cell: 2 μM [51], 12.5 μM [52]
		**Genotoxicity**	
		Oxidative DNA damage	Cell: 100 μM [53], 200 μM [54] DNA: 5 μM [54]
		**Hepatotoxicity and gastrointestinal toxicity**	
		Gastrointestinal tract and liver lesion in rats and mice treated with green tea extract containing EGCG (48.4%)	Gastrointestinal tract lesion: 1000 mg/kg/day (male and female rats) [55] Liver lesion: 1000 mg/kg/day (male and female rats) [55], 300 mg/kg/day (male mice) [55]
		High dose intake-associated liver damage in humans	Human: 704 mg/day [56]
	Gallic acid	**Cytotoxicity**	
		Cytotoxicity linked with ROS generation	Cell: 74 μM [57], 294 μM [58,59]
		**Genotoxicity**	
		Oxidative DNA damage	DNA: 5 μM [60], 200 μM [61]
		**Hepatotoxicity and nephrotoxicity**	
		Liver damage in mice and rats, and renal injury in rats	Liver damage: 200 mg/kg/day (male mice) [62], 100 mg/kg/day (male rats) [63] Renal injury: 100 mg/kg/day (male rats) [63]

Aβ: amyloid β, ROS: reactive oxygen species, EGCG: epigallocatechin gallate.

## Abbreviations

AD	Alzheimer’s disease
Aβ	amyloid β
APP	amyloid precursor protein
ROS	reactive oxygen species
EGCG	epigallocatechin gallate
NMR	nuclear magnetic resonance
8-oxodG	8-oxo-7,8-dihydro-2′-deoxyguanosine
PAINS	pan-assay interference compound
CYP	cytochrome P450
HOMO energy	highest occupied molecular orbital energy
